# Three-Factor Structure of the eHealth Literacy Scale Among Magnetic Resonance Imaging and Computed Tomography Outpatients: A Confirmatory Factor Analysis

**DOI:** 10.2196/humanfactors.9039

**Published:** 2018-02-19

**Authors:** Lisa L Hyde, Allison W Boyes, Tiffany-Jane Evans, Lisa J Mackenzie, Rob Sanson-Fisher

**Affiliations:** ^1^ Health Behaviour Research Collaborative, School of Medicine and Public Health Faculty of Health and Medicine University of Newcastle Callaghan Australia; ^2^ Priority Research Centre for Health Behaviour University of Newcastle Callaghan Australia; ^3^ Hunter Medical Research Institute New Lambton Heights Australia; ^4^ Hunter Cancer Research Alliance Newcastle Australia

**Keywords:** eHealth, literacy, factor analysis, measures, psychometrics

## Abstract

**Background:**

Electronic health (eHealth) literacy is needed to effectively engage with Web-based health resources. The 8-item eHealth literacy scale (eHEALS) is a commonly used self-report measure of eHealth literacy. Accumulated evidence has suggested that the eHEALS is unidimensional. However, a recent study by Sudbury-Riley and colleagues suggested that a theoretically-informed three-factor model fit better than a one-factor model. The 3 factors identified were awareness (2 items), skills (3 items), and evaluate (3 items). It is important to determine whether these findings can be replicated in other populations.

**Objective:**

The aim of this cross-sectional study was to verify the three-factor eHEALS structure among magnetic resonance imaging (MRI) and computed tomography (CT) medical imaging outpatients.

**Methods:**

MRI and CT outpatients were recruited consecutively in the waiting room of one major public hospital. Participants self-completed a touchscreen computer survey, assessing their sociodemographic, scan, and internet use characteristics. The eHEALS was administered to internet users, and the three-factor structure was tested using structural equation modeling.

**Results:**

Of 405 invited patients, 87.4% (354/405) were interested in participating in the study, and of these, 75.7% (268/354) were eligible. Of the eligible participants, 95.5% (256/268) completed all eHEALS items. Factor loadings were 0.80 to 0.94 and statistically significant (*P*<.001). All reliability measures were acceptable (indicator reliability: awareness=.71-.89, skills=.78-.80, evaluate=.64-.79; composite reliability: awareness=.89, skills=.92, evaluate=.89; variance extracted estimates: awareness=.80, skills=.79, evaluate=.72). Two out of three goodness-of-fit indices were adequate (standardized root mean square residual (SRMR)=.038; comparative fit index (CFI)=.944; root mean square error of approximation (RMSEA)=.156). Item 3 was removed because of its significant correlation with item 2 (Lagrange multiplier [LM] estimate 104.02; *P*<.001) and high loading on 2 factors (LM estimate 91.11; *P*<.001). All 3 indices of the resulting 7-item model indicated goodness of fit (χ^2^_11_=11.3; SRMR=.013; CFI=.999; RMSEA=.011).

**Conclusions:**

The three-factor eHEALS structure was supported in this sample of MRI and CT medical imaging outpatients. Although further factorial validation studies are needed, these 3 scale factors may be used to identify individuals who could benefit from interventions to improve eHealth literacy awareness, skill, and evaluation competencies.

## Introduction

### Consumer eHealth Literacy is Critical to Maximizing the Benefits of eHealth

Technologically-enabled health care is important at both the patient and service level, given the increasing resource and timing pressures on the health care system [1], the digital transformation of health-related industries [2], and changing consumer expectations about their role in care [3]. Electronic health (eHealth) refers to the organization and delivery of health services and information using the internet and related technologies [4]. eHealth holds potential as a scalable form of service delivery that is accessible, low-cost, promotes patient empowerment, and enhances patient-provider information exchange [5]. However, to reap the possible benefits, patients must be eHealth literate [6]. eHealth literacy refers to an individual’s ability to seek, find, understand, and appraise health information from electronic sources, and apply the knowledge gained to addressing or solving a health problem [6]. Limited ability to seek, find, understand, and appraise electronic health information has been recognized as a key self-reported barrier to the utilization of the internet for health purposes [7]. The first step in identifying individuals who may benefit from improved eHealth literacy is the development of valid and reliable tools assessing this construct.

### The eHealth Literacy Scale Is a Standardized and Widely Used Measure

The eHealth literacy scale (eHEALS) was among the first and continues to be one of the most commonly used self-reported measures of eHealth literacy [8,9]. The scale comprises 8 items, which assess consumers’ combined knowledge, comfort, and perceived skills at finding, evaluating, and applying electronic health information to health problems [8]. Consistent with the current definition of eHealth [4], all eHEALS items are specific to health information access via the Internet, as opposed to other electronic forms of information provision (eg, Compact Disc Read-Only Memory [CD-ROM], computer games). The scale was developed to address the need for an easily self-administrable eHealth literacy measure that could be applied across a wide range of populations and contexts [8]. Widespread adoption of the scale has been demonstrated, with the measure translated into multiple languages [10-17] and used across participants with diverse sociodemographic [10,15,16,18], ethnic [11,14,19], and disease profiles [13,20,21]. Items were originally developed and validated among Canadian youths more than a decade ago [8], and subsequent studies have demonstrated test-retest reliability across younger [14] and older age cohorts [10], internal consistency across populations of varying age and ethnicity [10,11,14,15,19,22], and measurement invariance across English-speaking countries [23]. However, inconsistent findings exist regarding the convergent and predictive validity of the scale [10,11,24], and debate continues about its factor structure [10-17,22,23,25-28]. We sought to contribute to this knowledge by assessing the factorial validity and internal consistency of a three-factor structure of the eHEALS.

### The Factor Structure of the eHealth Literacy Scale Is Uncertain

Norman and Skinner’s original factorial validation of the eHEALS found that the scale assesses a single dimension [8]. Numerous studies with the general public have supported this finding [10,11,14-16,22,25,26], including those specific to children [15], university students [14,16], and older adults [10,22]. However, the strength of these conclusions is limited by the common use of exploratory factor analysis (EFA) [8,10,11,14,15,22,25,26]. EFA originates from classical test theory and holds value in the early stages of scale development when factor structure is unknown and latent variable structures need to be identified [29]. EFA does, however, have some limitations. For example, it often involves subjective decision-making processes and does not account for the theory which may inform latent variable structures [30].

Confirmatory factor analysis (CFA) is an alternative analysis technique, also derived from classical test theory, which allows models to be tested via theoretically or empirically-driven hypotheses [31]. However, studies assessing a unidimensional eHEALS structure using CFA commonly report poor fit indices [13,23,27,28]. This may be because a single factor structure does not account for the multifaceted nature of the concept of eHealth literacy, such as its inherent literacy types (ie, traditional, health, information, scientific, media, and computer) or the multiple components of information retrieval and use (ie, finding, applying and evaluating electronic health information) [6]. Paige and colleagues [13] completed one of the only studies of the construct validity of the eHEALS using CFA with chronically ill patients and found evidence for a three-factor structure. Despite this, multidimensionality of the eHEALS was refuted on the basis that a large proportion of variance loaded on one factor only. The authors applied the partial credit model, which is a unidimensional item response theory technique, to conclude that a single structure exists, despite CFA values indicating a poor unidimensional fit [13]. A two-factor model based on the concepts of information-seeking and appraisal has also been tested [12,27,28]. Although this model has a strong theoretical basis, 2 of the 3 studies testing this structure reported inadequate fit indices [12,27]. Furthermore, all were based on translated versions of the scale, which can result in varied item meaning and interpretation [32].

### Recent Literature Proposes That the eHealth Literacy Scale Has a Three-Factor Structure

Sudbury-Riley and colleagues [23] used CFA to test a three-factor structure of the English-language version of the eHEALS with a multinational sample of adult internet users from the United Kingdom (n=407), New Zealand (n=276), and the United States (n=313). A hypothesis-driven approach was adopted, whereby 2 eHEALS items were mapped to an “awareness” factor, 3 items to a “skills” factor, and 3 items to an “evaluate” factor. These factors were derived from the self-efficacy and social-cognitive theoretical constructs underpinning eHealth literacy [8,23]. Self-efficacy theory is based on the premise that goal achievement is mediated by self-belief and confidence, and social cognitive theory states that social context influences goal achievement [33]. Sudbury-Riley and colleagues [23] therefore proposed that an individual’s awareness is shaped by their environment (eg, exposure to Web-based health information), their skills are influenced by social factors (eg, modeling, instruction, and social persuasion), and their ability to evaluate eHealth resources is mediated by their confidence and persistence. CFA fit indices supported the hypothesized three-factor eHEALS structure across all 3 countries [23].

### Further Research Is Needed to Verify the Three-Factor Structure of the Standardized eHealth Literacy Scale With Patient Populations

Sudbury-Riley and colleagues’ [23] study contributes to our understanding of the underlying structures of the eHEALS, however, it has some limitations. In particular, a modified version of the scale was used, based on feedback from the authors’ family, friends, and colleagues, in which “and information” was added to items to address the increasing interactivity of eHealth materials. It is therefore unclear whether the three-factor structure also applies to the original version of the scale. The study was also conducted with middle-aged members of the general population, restricting the generalizability of findings across medical populations and age cohorts. This adds to the common underrepresentation of chronically ill patients in the eHEALS measurement literature, despite the potential benefits of eHealth to this population [13].

Given that evidence about the properties of a measure is accumulated over a number of studies, the appropriate next step it is to determine whether Sudbury-Riley and colleagues’ [23] findings can be replicated in a different population. To address this need, and also overcome some of the limitations of Sudbury-Riley and colleagues’ work [23], this factorial validation study was conducted with patients, using the standardized eHEALS. Magnetic resonance imaging (MRI) and computed tomography (CT) medical imaging outpatients represent a high volume of patients with diverse demographic characteristics and medical diagnoses [34,35], and as such, research completed with these patients may have high generalizability. Furthermore, MRI and CT medical imaging outpatients require substantial preparatory information that could potentially be delivered online [36]. Hence, this study aimed to test the factorial validity and internal consistency of the three-factor structure of the eHEALS, identified by Sudbury-Riley and colleagues [23], among MRI and CT medical imaging outpatients.

## Methods

### Design and Setting

A cross-sectional survey of CT and MRI medical imaging outpatients was conducted in a medical imaging clinic at a tertiary referral hospital located in regional New South Wales, Australia.

### Participants

Eligible participants were attending for an outpatient CT or MRI appointment at the tertiary referral hospital, were 18 years or older, and had access to the internet for personal use. Participants were excluded from the study if they had a cognitive or physical impairment that precluded them from providing informed consent or participating in the study, or if they were unable to complete the questionnaire because of poor English proficiency. These criteria mean that a diversity of participants in terms of frequency, confidence, and reasons for personal use of the internet were eligible to participate. Consistent with the original eHEALS validation study [8], use of the internet for health was not an eligibility requirement.

### Procedure

Patients who were potentially eligible for the study were identified by medical imaging reception staff when they presented for their outpatient appointment. These patients were informed about the research and invited to speak with a trained research assistant. Interested patients were provided with a written information sheet and introduced to the research assistant, who gave an overview of the study and obtained patients’ verbal consent to participate. The age, gender, and scan type of noninterested and nonconsenting patients were recorded. Consenting participants were provided with a tablet computer and asked to complete a Web-based questionnaire before their scan. A paper version of the questionnaire was provided to participants who requested it. Ethics approval was obtained from the Hunter New England Human Research Ethics Committee (16/10/19/5.11) and University of Newcastle (H-2016-0386).

### Measures

Participants’ eHealth literacy was assessed using the 8-item English-language version of the eHEALS [8]. Respondents indicated their level of agreement with each statement on a 5-point Likert scale, which was scored from 1 “strongly disagree” to 5 “strongly agree.”

Sociodemographic, scan, and information preference characteristics were examined using standard items. These items assessed participant age, gender, marital status, highest level of education completed, postcode, and scan type. Postcodes were mapped to the Accessibility/Remoteness Index of Australia Plus 2011 classification to examine remoteness [37] and categorized as metropolitan (major cities of Australia) or nonmetropolitan (inner regional, outer regional, remote, or very remote Australia). One item, adapted from an existing health information wants questionnaire [38], assessed how much information participants liked to have about their health. Response options were “no information,” “some information,” and “a lot of information.”

**Figure 1:**
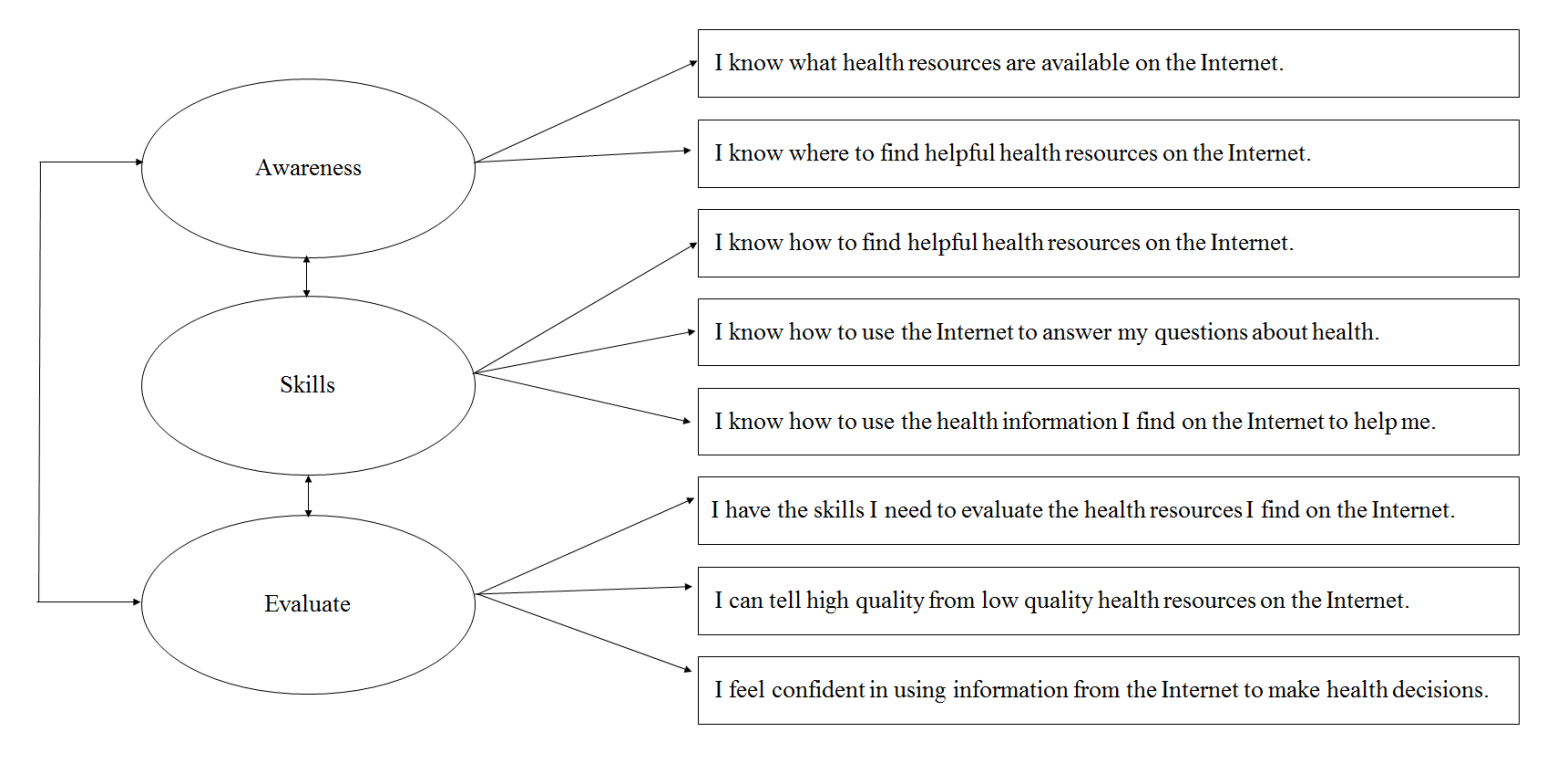
eHealth Literacy Scale three-factor model proposed by Sudbury-Riley and colleagues.

Internet characteristics were assessed by 2 items. Use of the internet for scan preparation was assessed by an author-developed item: *Have you searched the internet for information to help you prepare for your scan?* with response options “no,” “yes,” and “don’t know.” Frequency of internet use was measured with a single item used in existing informatics literature [39], in which participants respond on a 6-point scale ranging from “less than once a month” to “several times a day.”

### Sample Size

Rules of thumb for CFA recommend a sample size of at least 200 participants [40,41] or 10 participants per parameter estimated [42]. Wolf and colleagues [43] found that a sample size of at least 150 is required for three-factor models with fewer than 4 indicator variables per factor and assuming strong factor loadings of 0.80. To accommodate deviation from these assumptions, and given that 19 parameters were estimated for the eHEALS CFA, the more conservative estimate of at least 200 participants was applied to this study.

### Statistical Analyses

Participant characteristics and eHEALS responses were summarized as frequencies and percentages, or means and standard deviations. Consent bias was assessed for gender, scan type, and age group using chi-square tests. CFA was undertaken using the CALIS procedure of SAS software v9.4 (SAS Institute, Cary, NC, USA). We chose CFA as it is the same theoretically-sound technique used by Sudbury-Riley and colleagues [23] and therefore allowed for a direct comparison of results. Given the high completion rate (98.1% [256/261] of participants who started the eHEALS completed all items), this analysis was restricted to participants with complete eHEALS data. The relationship between latent variables (ie, awareness, skills, evaluate) and manifest variables (eHEALS items 1-8), as proposed by Sudbury-Riley and colleagues [23], was tested using structural equation modeling (Figure 1). All loadings were standardized, with variances fixed at 1. The model was estimated using the full information maximum likelihood method. Standardized factor loadings and covariances were calculated with 95% CIs.

Reliability measures included indicator reliability to determine the percentage of variation in the item explained by each factor, composite reliability to assess internal consistency (>.70 ideal) [29], and variance extracted estimates (VEEs) to determine the amount of variance captured by factors with regard to variance attributable to measurement error (>.50 ideal) [44]. Discriminant validity was assessed following the method proposed by Anderson and Girbing [45].

Model goodness of fit was assessed using a range of metrics. Absolute indices included the chi-square statistic, the chi-square to degrees of freedom ratio (<2 ideal) [46], and the standardized root mean square residual (SRMR; <.055 ideal) [29]. The incremental index was reported as the comparative fit index (CFI; >.95 good fit) [47]. The parsimony index used was the root mean square error of approximation (RMSEA; <.05 close approximate fit, .05-.08 acceptable fit, >.10 poor fit) [29,47]. Lagrange multiplier (LM) estimates of items on different factors were assessed to identify complex items and possible ways to improve the model.

## Results

### Sample

A total of 405 potentially eligible patients were invited to discuss the study with a research assistant during the 7-week recruitment period. Of the invited patients, 87.4% (354/405) were interested in participating in the study, and of these, 75.7% (268/354) were eligible. Of these eligible participants, 97.4% (261/268) started the eHEALS, and 95.5% (256/268) completed all eHEALS items. There were no significant differences between patients who were and were not interested in participating in the study based on gender, scan type, or age group. Table 1 provides a summary of the sociodemographic, scan, and internet characteristics of eligible participants. Multimedia Appendix 1 provides a summary of participant responses to eHEALS items.

### Confirmatory Factor Analysis

Convergence between the implied and observed variance covariance matrices was achieved within 10 iterations. As shown in Table 2, all factor loadings were at or above 0.80 and were statistically significant (*P*<.001). All CRs exceeded .70, indicating good reliability, and all VEEs exceeded the cutoff of .50, indicating convergent validity. Discriminant validity of the model was demonstrated, with statistically significant chi-square difference-tests (*P*<.001) for each pair of factors. The absolute index SRMR was .038, indicating adequate fit to the hypothesized model. The incremental index CFI was .944 and therefore close to the .95 threshold of acceptability (Table 3). However, the chi-square statistic (χ^2^_17_=124.2) was highly significant and suggestive of poor fit, and the chi-square statistic to degrees of freedom ratio of 7.3 exceeded the acceptability cutoff of 2 [46]. The parsimony index RMSEA was .16, indicating poor fit.

**Table 1:** Participant sociodemographic, scan, and internet characteristics (N=268).

Characteristic		n (%)^a^
Mean age years (SD)		53 (15)
**Gender**		
	Male	120 (44.8)
	Female	148 (55.2)
**Marital status**		
	Married or partner	148 (64.9)
	Not married/living with partner	80 (35.1)
**Education completed**		
	High school or less	169 (63.1)
	More than high school	99 (36.9)
**Geographic location**		
	Metropolitan	212 (79.1)
	Nonmetropolitan	56 (20.9)
**Scan type**		
	CT	104 (38.8)
	MRI	160 (59.7)
	Don’t know	4 (1.5)
**Used internet for scan**		
	Yes	29 (10.9)
	No	237 (88.8)
	Don’t know	1 (0.3)
**Frequency of internet use**		
	Less than once a month	11 (4.1)
	Once a month	5 (1.8)
	A few times a month	14 (5.2)
	A few times a week	36 (13.5)
	About once a day	51 (19.1)
	Several times a day	150 (56.2)
**Information amount preference**		
	No information	2 (0.8)
	Some information	59 (26.0)
	A lot of information	166 (73.1)

^a^Number of observations for each characteristic may not total 268 because of missing data.

**Table 2:** Factor loading and residual error estimates for confirmatory factor analysis of hypothesized model.

Factor-variable		Factor loadings (95% CI)	Error estimates (95% CI)	IR^a^	CR^b^	VEE^c^
**Awareness**						
	I know what health resources are available on the Internet	0.85 (0.80-0.89)^d^	0.29 (0.21-0.36)^d^	.71	.89	.80
	I know where to find helpful health resources on the Internet	0.94 (0.91-0.97)^d^	0.11 (0.05-0.17)^d^	.89		
**Skills**						
	I know how to find helpful health resources on the Internet^e^	0.90 (0.86-0.93)^d^	0.20 (0.14-0.26)^d^	.80	.92	.79
	I know how to use the internet to answer my questions about health	0.88 (0.85-0.92)^d^	0.22 (0.16-0.28)^d^	.78		
	I know how to use the information I find on the internet to help me	0.88 (0.85-0.92)^d^	0.22 (0.16-0.28)^d^	.78		
**Evaluate**						
	I have the skill I need to evaluate the health resources I find on the Internet	0.89 (0.85-0.92)^d^	0.21 (0.15-0.28)^d^	.79	.89	.72
	I can tell high quality from low quality health resources on the Internet	0.86 (0.82-0.90)^d^	0.26 (0.19-0.33)^d^	.74		
	I feel confident in using information from the internet to make health decisions	0.80 (0.75-0.85)^d^	0.36 (0.28-0.44)^d^	.64		

^a^IR: indicator reliability.

^b^CR: composite reliability.

^c^VEE: variance extracted estimate.

^d^*P*<.001.

^e^This item was dropped in the alternative 7-item model.

**Table 3:** Goodness-of-fit indices for tested models.

Index type and fit index		Statistics for hypothesized 8-item model	Statistics for tested 7-item model
**Absolute index**			
	Chi-square	124.2	11.3
	Chi-square degrees of freedom	17	11
	*P*-value for the chi-square statistic	<.001	.417
	SRMR^a^	.038	.012
**Incremental index**				
	Bentler CFI^b^	.944	.999
**Parsimony index**				
	RMSEA^c^ estimate	.156	.011
	RMSEA lower 90% CI	.131	.000
	RMSEA upper 90% CI	.182	.066

^a^SRMR: standardized root mean square residual.

^b^CFI: comparative fit index.

^c^RMSEA: root mean square error of approximation.

When investigating the possible reasons for less than ideal fit, LM estimates provided strong evidence for a path between item 3 “I know how to find helpful health resources on the Internet” and the awareness factor (LM estimate 107.66; *P*<.001). There was also strong evidence for a path between item 2 “I know where to find helpful health resources on the Internet” and item 3 “I know how to find helpful health resources on the Internet” (LM estimate 91.11; *P*<.001). Given apparent overlap between items 2 and 3, a 7-item model which excluded item 3 was tested, which indicated good model fit (Table 3). See Multimedia Appendix 2 for factor loading and residual error estimates for this altered model.

## Discussion

### Principal Findings

This study was the first to examine the theoretically-derived three-factor structure of the eHEALS, as proposed by Sudbury-Riley and colleagues [23], among a sample of MRI and CT medical imaging outpatients. This three-factor structure was supported, with 2 out of 3 goodness-of-fit indices indicating adequate fit to the hypothesized model. Although these findings oppose accumulated evidence for a unidimensional structure of the eHEALS [8,10,11,14-16,22,25,26], they are consistent with the social cognitive and self-efficacy theory underpinning eHealth literacy [8,23,33]. As a result, it may be timely for researchers to examine patients’ eHealth literacy across eHEALS factors to inform targeted eHealth literacy improvement interventions. This study contributes important knowledge about the structure of the eHEALS, yet further factorial analyses, including multidimensional item response theory analyses, are required across populations to increase the reliability of these findings.

#### Findings Broadly Support the Proposed Three-Factor Structure of the eHEALS

The proposed model demonstrated strong internal consistency and discriminant validity, suggesting that items within each factor measured the same general construct, and these constructs were sufficiently different from one another. Similarly, 2 out of 3 fit indices demonstrated good fit to the proposed three-factor model. Factor loadings were high and statistically significant, similar to that reported by Sudbury-Riley and colleagues [23]. This finding contrasts to the majority of existing literature, where it is argued that a single factor structure exists [8,10-16,19,22,25,26]. Most such prior research is based on data-driven EFA techniques [8,10,11,14,15,22,25,26], which may indicate that limited reference to the theoretical underpinnings of eHealth literacy has resulted in inaccurate interpretations of eHEALS data in the past.

#### Not all Goodness-of-Fit Indices Were Ideal

Poor fit of the parsimony index suggests that complexity exists within the three-factor model. RMSEA estimates have also been identified as a poor performing goodness-of-fit metric in other CFA eHEALS literature [12,13,27] and are rarely reported as being a close approximate fit, indicating that relationships among items need to be interrogated. When we investigated further, it was found that item 3 “I know how to find helpful health resources on the Internet” loaded on both “skills” and “awareness” domains, and correlated significantly with item 2 “I know where to find helpful health resources on the Internet.” This finding supports that of Sudbury-Riley and colleagues [23], who identified substantial overlap between items 2 and 3. Potential item homogeneity is also evident in prior literature, as measures of internal consistency have commonly been reported to be approaching the .95 threshold of acceptability for Cronbach alpha [10,11,15,19], with some reported to have reached .97 [22]. The redundancy of items 2 and 3 is unsurprising, given their similar structure and meaning (ie, about how and where to find helpful health resources on the Internet). It is also possible that the low education level of the sample [48], and the distressing setting of a hospital waiting room [49], contributed to participants’ difficulties in differentiating between item meanings. However, patient understanding of eHEALS items has been questioned previously, and the need for further research investigating item interpretation across populations has been indicated [11].

For this study, we did not restrict our sample to health-related internet users. This aligns with the majority of studies assessing the factorial validity of the eHEALS, including Norman and Skinner’s original validation study [8,10-17,19,22,26-28]. Furthermore, Norman and Skinner [8] highlight the potential application of the scale to those with varying levels of technology use. eHEALS response options of *disagree* and *strongly disagree* provide for those who do not use the internet for health. Despite this, some participants within this study voluntarily reported being unsure of how to respond to each item as they did not use the internet for health. This anecdotal feedback suggests that items within the scale may not be interpretable to the wide population for which it was originally intended [8], and further research is needed to investigate the face and content validity of the scale among those who do and do not use the internet for health purposes.

As model fit improved when item 3 “I know how to find helpful health resources on the Internet” was excluded, an adapted 7-item eHEALS may be appropriate to consider. Reducing the number of items would result in two factors containing 2 items, which could create difficulties with model identification and convergence [29]. Likewise, it is unknown whether a reduced 2-item “skill” factor would adequately measure the construct and appropriately detect changes over time. As such, further research is needed to test the psychometric properties (specifically content validity, test-retest reliability, predictive validity, and responsiveness) of a 7-item eHEALS. Until this point, it is recommended that the standardized 8-item scale is used, with consideration of preliminary evidence supporting a three-factor structure.

#### The Three-Factor Structure of the eHEALS May Reflect an eHealth Literacy Pathway Among internet Users

Despite some fit indices being less than ideal, considering eHealth literacy by factor may help to guide Web-based health information provision in research and clinical practice. Furthermore, in accordance with the eHealth literacy continuum proposed by Diviana and colleagues [12], the eHEALS may measure an eHealth literacy pathway. In this instance, eHEALS factors are structured sequentially, and a user gradually demonstrates proficiency in more complex tasks. That is, a user must first be aware of eHealth resources before they can use their skills to navigate and interact with electronic content, and finally evaluate content quality and applicability to their health situation. Only once a user has undertaken all 3 of these steps, will they be able to effectively engage with eHealth resources and reap related benefits. This proposed pathway structure is supported by findings of Neter and colleagues [24], who reported that success rates gradually declined for older adults performing health-related computerized simulation tasks, as they stepped through the process of accessing, understanding, appraising, applying, and generating new health information. These findings may, however, be influenced by order effects of the simulated tasks [50], and further research is needed to validate such a causal pathway.

#### Important Implications for the Future Development and Evaluation of eHealth Literacy Improvement Strategies

On the basis of these findings, researchers and health care professionals have the opportunity to identify areas (ie, awareness, skills, or evaluate) where competency is low and target eHealth literacy improvement interventions accordingly. These interventions may, for example, include clinician recommendations to Web-based materials to increase awareness and reduce the need to evaluate content [51], training sessions to enhance eHealth literacy skills [52], or the promotion of checklists to aid in the evaluation of Web-based resources [53]. Additionally, user characteristics such as sociodemographic, health, and Internet use attributes that are associated with lower competency across eHEALS factors could be identified, so that assistance is directed toward those most in need. No studies have been conducted to determine the competency of individuals across eHEALS awareness, skill, and evaluate domains, and further research is needed.

### Limitations

CFA was selected as it represents an understudied yet rigorous aspect of classical test theory and logically extends on the existing body of EFA and CFA measurement literature. The recent emergence of item response theory analyses of the eHEALS [12,13,16] has advantages over classical test theory approaches, including the capacity to establish increased item level psychometric information (eg, item difficulty). The application of multidimensional item response theory techniques to validate the three-factor eHEALS structure should be explored further. Furthermore, this study assessed one psychometric property (ie, factorial validity), and more research is needed to investigate other understudied measurement properties of the eHEALS, such as its predictive validity.

It is possible that findings may not be generalizable beyond the medical imaging context. Similarly, as most participants reported using the internet at least daily (75.3%, 201/267), study findings may not be generalizable to those who use the internet less frequently. As we did not ask participants about the activities they undertook online, it is unclear whether the results are applicable to those who do or do not use the internet for health. Future research is consequently needed to validate study findings across patients with diverse demographics, medical diagnoses, and internet use patterns. Additionally, our study was based on the standardized version of the eHEALS. As recognized in prior research [12,23], this version may not sufficiently capture competency in using Web 2.0 (eg, social networking) for health. Further research is needed to determine whether scale modifications are needed to reflect the evolving nature of eHealth interventions.

### Conclusions

Although potential item redundancy impacted fit indices, the three-factor structure of the eHEALS was broadly supported. On the basis of these findings, the eHEALS could be used to inform the development of tailored eHealth literacy enhancement strategies, which may in turn increase engagement with Web-based health resources. Further research is needed to confirm the three-factor structure across other medical settings and populations to support the generalizability of these findings.

## Appendix

Multimedia Appendix 1 Participant responses to eHEALS items (n=261).

Multimedia Appendix 2 Factor loading and residual error estimates for the confirmatory factor analysis of the 7-item model.

## Abbreviations

CFA: confirmatory factor analysis
CFI: comparative fit index
CT: computed tomography
EFA: exploratory factor analysis
LM: Lagrange multiplier
eHEALS: 8-item eHealth literacy scale
eHealth: electronic health
MRI: magnetic resonance imaging
RMSEA: root mean square error of approximation
SRMR: standardized root mean square residual
VEE: variance extracted estimate
