# Supplementary material for: Three-Factor Structure of the eHealth Literacy Scale Among Magnetic Resonance Imaging and Computed Tomography Outpatients: A Confirmatory Factor Analysis
Source: JMIR Hum Factors. 2018 Feb 19;5(1):e6. doi: 10.2196/humanfactors.9039 (PMC5838360; doi:10.2196/humanfactors.9039)
Supplement: Multimedia Appendix 1 [file humanfactors_v5i1e6_app1.pdf]

Multimedia Appendix 1

Multimedia Appendix Table 1: Participant responses to eHEALS items (n = 261)

**Factor**<br>Variable	Strongly disagree	Disagree	Undecided	Agree	Strongly agree
	n (%)[a]				
**Awareness**					
I know what health resources are available on the internet	12 (4.6)	50 (19.2)	55 (21.1)	115 (44.1)	29 (11.1)
I know where to find helpful health resources on the internet	10 (3.8)	50 (19.2)	47 (18.0)	121 (46.4)	33 (12.6)
**Skills**					
I know how to find helpful health resources on the internet	9 (3.5)	41 (15.7)	37 (14.2)	135 (51.9)	38 (14.6)
I know how to use the internet to answer my questions about health	12 (4.7)	28 (10.9)	36 (14.0)	132 (51.2)	50 (19.4)
I know how to use the information I find on the internet to help me	10 (3.9)	34 (13.2)	48 (18.6)	126 (49.8)	40 (15.5)
**Evaluate**					
I have the skill I need to evaluate the health resources I find on the internet	14 (5.4)	48 (18.7)	53 (20.6)	93 (36.2)	49 (19.1)
I can tell high quality from low quality health resources on the internet	11 (4.3)	59 (23.0)	63 (24.6)	79 (30.9)	44 (17.2)
I feel confident in using information from the internet to make health decisions	21 (8.2)	52 (20.3)	72 (28.1)	81 (31.6)	30 (11.7)

[a] Number of observations for each eHEALS item may not total 261 due to missing data
