# Supplementary material for: Three-Factor Structure of the eHealth Literacy Scale Among Magnetic Resonance Imaging and Computed Tomography Outpatients: A Confirmatory Factor Analysis
Source: JMIR Hum Factors. 2018 Feb 19;5(1):e6. doi: 10.2196/humanfactors.9039 (PMC5838360; doi:10.2196/humanfactors.9039)
Supplement: Multimedia Appendix 2 [file humanfactors_v5i1e6_app2.pdf]

Multimedia Appendix 2

Multimedia Appendix Table 2: Factor loading and residual error estimates for the confirmatory factor analysis of the 7-item model

**Factor**<br>Variable	Factor loadings (95% CI)	Error estimates (95% CI)	IR[a]	CR[b]	VEE[c]
**Awareness**					
I know what health resources are available on the internet	0.85 (0.80 to 0.89)[e]	.28 (.20 to .36)[e]	.72	.89	.80
I know where to find helpful health resources on the internet	0.94 (0.90 to 0.98)[e]	.12 (.05 to .19)[f]	.89		
**Skills**					
I know how to use the internet to answer my questions about health	0.90 (0.86 to 0.93)[e]	.20 (.14 to .25)[e]	.80	.90	.82
I know how to use the information I find on the internet to help me	0.92 (0.89 to 0.94)[e]	.16 (.11 to .22)[e]	.84		
**Evaluate**					
I have the skills I need to evaluate the health resources I find on the internet	0.89 (0.86 to 0.93)[e]	.21 (.14 to .27)[e]	.79	.89	.72
I can tell high quality from low quality health resources on the internet	0.86 (0.82 to 0.90)[e]	.26 (.19 to .33)[e]	.74		
I feel confident in using information from the internet to make health decisions	0.80 (0.75 to 0.85)[e]	.36 (.28 to .44)[e]	.64		

[a] IR: Indicator Reliability
[b] CR: Composite Reliability
[c] VEE: Variance Extracted Estimate
[e] $P < .001$
[f] $P = .001$
